# SATMF Suppresses the Premature Senescence Phenotype of the ATM Loss-of-Function Mutant and Improves Its Fertility in *Arabidopsis*

**DOI:** 10.3390/ijms21218120

**Published:** 2020-10-30

**Authors:** Yi Zhang, Hou-Ling Wang, Yuhan Gao, Hongwei Guo, Zhonghai Li

**Affiliations:** 1 Beijing Advanced Innovation Center for Tree Breeding by Molecular Design, Beijing Forestry University, Beijing 100083, China; yizhang@bjfu.edu.cn (Y.Z.); whling@bjfu.edu.cn (H.-L.W.); 2 Key Laboratory of Pest Management in Crops of the Ministry of Agriculture, Institute of Plant Protection, Chinese Academy of Agricultural Sciences, Beijing 100193, China; gaoyuhanamy@gmail.com; 3 Key Laboratory of Molecular Design for Plant Cell Factory of Guangdong Higher Education Institutes, Department of Biology, Southern University of Science and Technology (SUSTech), Shenzhen 518055, China

**Keywords:** leaf senescence, DNA damage, ATM, fertility, SATMF

## Abstract

Leaf senescence is the final stage of leaf development. It is accompanied by the remobilization of nutrients from senescent leaves to developing organs. The occurrence of senescence is the consequence of integrating intrinsic and environmental signals. DNA damage triggered by stresses has been regarded as one of the reasons for senescence. To prevent DNA damage, cells have evolved elaborate DNA repair machinery. The ataxia telangiectasia mutated (ATM) functions as the chief transducer of the double-strand breaks (DSBs) signal. Our previous study suggests that ATM functions in lifespan regulation in *Arabidopsis*. However, ATM regulatory mechanism on plant longevity remains unclear. Here, we performed chemical mutagenesis to identify the components involved in ATM-mediated longevity and obtained three dominant mutants *satmf1~3*, *suppressor of atm in fertility*, displaying delayed senescence and restored fertility in comparison with *atm* mutant. Molecular cloning and functional analysis of SATMF (suppressor of atm in fertility) will help to understand the underlying regulatory mechanism of ATM in plants, and shed light on developing new treatments for the disease Ataxia-telangiectasia.

## 1. Introduction

Leaves are the primary photosynthetic organs that use the photosynthetic system to fix CO_2_ to produce carbohydrates, thus providing energy for plant growth and development [1]. Leaf senescence is an inevitable physiological process, accompanied by the nutrient remobilization from senescent leaves to young leaves or other developmental organs [2,3,4]. Although leaf senescence is a genetically controlled process, it can be induced by various endogenous and environmental stresses [4,5,6,7,8,9,10].

Cells have evolved elaborate DNA repair machinery to prevent DNA damage. A growing body of evidence indicates that the loss of DNA damage repair induces premature senescence in animals and plants [11,12]. Paradoxically, DNA repair can itself be subject to age-related changes and deterioration [13]. DNA damage repair efficiency also changes during aging, which influences mismatch repair (MMR), base excision repair (BER), nucleotide excision repair (NER), and double-strand break (DSB) repair. Most of the evidence supporting DNA damage-induced senescence comes from the fact that mutations in genes involved in DNA repair lead to multiple premature aging symptoms, supporting the idea that the balance between DNA damage and repair determines the rate of aging [13]. For example, WRN protein, mutated in Werner syndrome (WS) characterized by premature aging in young adults, is involved in both homologous recombination (HR) and non-homologous end joining (NHEJ) pathways [14]. Mutation in Lamin A causes Hutchinson Gilford progeria syndrome (HGPS) and also impairs HR [15]. Loss-of-function of the ATM (ataxia telangiectasia mutated) shortens lifespan due to cancer and ischemic heart disease [16,17], and ATM has been reported to be a potential target for alleviating senescence through regulating lysosomal acidification [18]. In eukaryotes, the initial stages of the response to DNA damage are governed by two closely related protein kinases, ATM and ATR (ATM- and RAD3-related) [16]. ATM is activated by auto/transphosphorylation in response to DSBs and leads to the activation of cell cycle checkpoints, DNA repair or apoptosis, while ATR is generally activated by persisting single-stranded DNA breaks (SSBs) [16,19].

Like other eukaryotes, plants have the orthologs of ATM/ATR [20,21]. Functional analyses of ATM or ATR by using knockout mutants reveal that they display overlapping and distinct roles in regulating plant development and in response to DNA damage. Compared with *atr* mutants, the *atm* mutants in *Arabidopsis* are supersensitive to DSB-inducing agents such as methyl methane sulphonate (MMS) or γ-irradiation [22]. Reverse genetic screening revealed that loss of ATM function by T-DNA insertion or application of a specific kinase inhibitor promotes DSBs-induced and age-dependent leaf senescence, suggesting that ATM is functionally conserved in animals and plants [12]. Moreover, DSBs-regulated genes were overlapped with age-regulated genes, indicating that DSBs induce similar changes on transcript profile with the natural senescence process [12], further supporting the regulatory role of DSBs in aging [11,23]. 

Our previous work revealed that DSBs increase and DNA repair efficiency decreases as a leaf is aging, and DSBs alone could induce premature senescence, demonstrating the correlation between DSBs and leaf senescence. The PI3K-like protein kinase ATM is involved in repairing age-dependent DNA damage, and the deficiency in ATM resulted in early senescence [12]. Nevertheless, how ATM regulates leaf senescence is still unclear. In this study, we generated transgenic plants overexpressing *ATM*, which suppressed the early senescence phenotypes of *atm* mutant. To identify the components involved in ATM-mediated longevity, we performed chemical mutagenesis by treating *atm* seeds with ethyl methanesulfonate (EMS) [24], and obtained three suppressor lines (*samtf1~3*, suppressor of *atm* in fertility), which displayed delayed senescence phenotypes upon treatment with bleomycin (BLM), an inducer of DSBs. Molecular cloning and functional analysis of SATMF (suppressor of atm in fertility) will contribute to understanding the underlying mechanism of ATM in leaf senescence.

## 2. Results and Discussion

### 2.1. Overexpression of ATM Delays Leaf Senescence

Our previous study showed that ATM expression levels decrease as leaf aging and loss-of-function of ATM accelerates leaf senescence process [12]. To further assess whether the expression of ATM mRNA is due to transcriptional regulation, we generated transgenic *Arabidopsis* plants expressing a GUS (β-glucuronidase) gene driven by the *ATM* promoter containing a 3 kb fragment upstream of the start codon (*pATM:GUS*/Col-0). GUS staining of 8-, 16- and 48-d-old *pATM:GUS*/Col-0 plants showed higher GUS staining in young leaves than in mature or old leaves (Supplementary Figure S1), indicating that ATM transcription declines during plant leaf aging.

**Figure 1:**
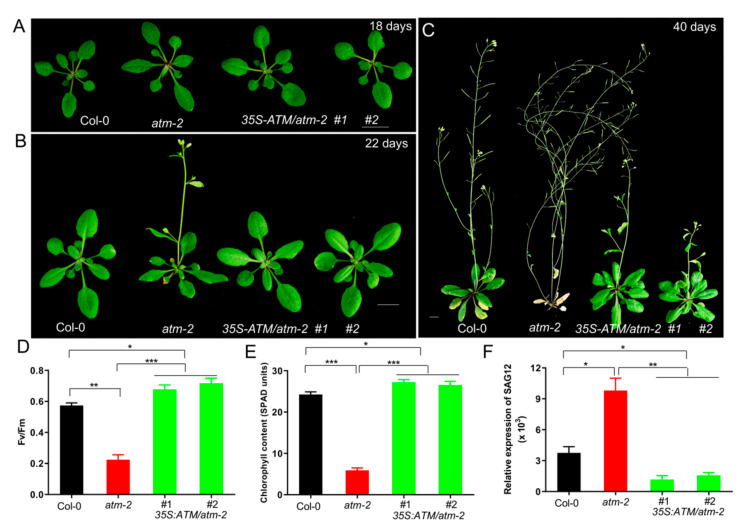
Overexpression of *ATM* (ataxia telangiectasia mutated) suppresses the early senescence phenotypes of *atm* mutant. (**A**) Rosette leaves of 18-day-old plants of Col-0, *atm-2* and *35S-ATM/atm-2*. (**B**) Overexpression of *ATM* suppresses the early flowering phenotype of *atm-2* mutant under long-day conditions (18 L/6 D). (**C**) Senescence phenotypes of 40-day-old plants of Col-0, *atm-2* and *35S-ATM/atm-2*. (**D**–**F**) Photochemical efficiency of PSII (Fv/Fm) (**D**), chlorophyll contents (**E**), and qRT-PCR analysis of *SAG12* expression (**F**) in the third and fourth leaves of 35-day-old plants of Col-0, *atm-2* and *35S-ATM/atm-2*. Three biological replicates were performed. Error bars represent standard deviation (SD, *n* = 24 for **D** and **E**; *n* = 3 for **F**). Student’s *t* test, * *p* < 0.05, ** *p* < 0.01 and *** *p* < 0.001.

We next tested whether constitutive overexpression of *ATM* could delay leaf senescence. Towards this end, we generated a transgenic line overexpressing *ATM* under the control of the 35S promoter in Col-0 and *atm* backgrounds, respectively. Interestingly, we found that overexpression of *ATM* in wild-type Col-0 background (*35S:ATM/*Col-0) caused gene co-suppression (~75%), leading to *atm*-like senescence phenotypes, which is not observed in *35S:ATM/atm* plants. We examined the senescence phenotypes of the *atm* and *35S:ATM/atm* at different developmental stages. Overexpression of *ATM* restored multiple abnormal phenotypes of *atm* mutants, such as small leaves (Figure 1A), slightly early flowering (Figure 1B), and premature leaf senescence (Figure 1C). Compared with Col-0, rosette leaves of the 40-d-old *atm* mutant exhibited severe yellowing, whereas *35S:ATM/atm* leaves remained green, suggesting further that ATM is a negative regulator of leaf senescence [12]. We next performed a detailed examination of senescence phenotypes in the third and fourth leaves of wild-type Col-0, *atm* and *35S:ATM/atm* plants. Photochemical efficiency of photosystem II (PSII, Fv/Fm) and chlorophyll contents in *atm* leaves were significantly less than that in Col-0 and *35S:ATM/atm* leaves (Figure 1D,E). In contrast, expression of *senescence-associated gene 12* (*SAG12*), a senescence marker gene, in the *atm* mutant was more dramatically induced in comparison with Col-0 and *35S:ATM/atm* (Figure 1F). Together, these results suggest that overexpression of *ATM* delays leaf senescence.

### 2.2. A Genetic Screen of atm Mutant Suppressors

The above data demonstrate that ATM functions as a negative regulator of leaf senescence. However, how ATM regulates leaf senescence is still unknown. To identify the components involved in ATM-mediated plant longevity, we performed forward genetic screens to identify the suppressors of *atm* mutant. Loss of ATM function causes multiple phenotypes, including early flowering, early senescence, decreased fertility, and increased plant height (Supplementary Figure S2A). Given that numerous environmental stresses such as shade or drought accelerate leaf senescence [4], while the fertility phenotype is relatively stable and easy to observe, we sought to identify the suppressors with restored fertility but not restored senescence phenotypes, termed as *suppressor of atm in fertility* (*satmf*). We assumed that the premature leaf senescence phenotypes of *atm* were suppressed along with the restoration of fertility. To this end, we carried out chemical mutagenesis by treating the seeds of wild-type *Arabidopsis* Col-0 and *atm* mutant with EMS [24]. Given that *atm* mutant is more sensitive to DNA-damage reagents such as EMS than wild-type Col-0 [20], seeds of Col-0 and *atm-2* were treated with serial concentrations of EMS (0.2% for 15 h, 0.3% for 12 h, and 0.4% 10 h) to seek for the optimal mutagenesis conditions (Supplementary Figure S2B). We found that mutagenesis with 0.3% EMS for 12 h was suitable for *atm* seeds, which slightly affected seed germination (Supplementary Figure S2C), but produced various traits.

**Figure 2:**
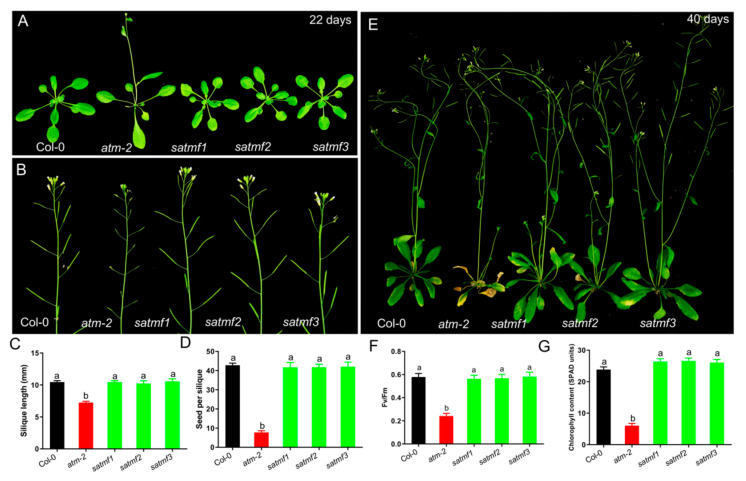
SATMF suppresses the early senescence phenotypes of *atm* mutant and improves its fertility. (**A**) Rosette leaves of 22-day-old plants of Col-0, *atm-2*, *satmf1*, *satmf2* and *satmf3*. (**B**) Fertility of Col-0, *atm-2*, *satmf1*, *satmf2* and *satmf3* plants. Silique length (**C**), Number of seeds per silique (**D**), Senescence phenotypes (**E**) of 40-day-old plants of Col-0, *atm-2*, *satmf1*, *satmf2* and *satmf3*. (**F**) Photochemical efficiency of PSII (Fv/Fm) and chlorophyll contents (**G**) in the third and fourth leaves of 35-day-old plants of indicated genetic backgrounds. Different letters represent significant differences (two-way ANOVA and post-hoc Tukey test, *p* < 0.05).

The model plant *Arabidopsis* has been widely used for forward genetic screening due to its short lifecycle, relatively little genome and abundant genetic resources [25,26,27]. In *Arabidopsis*, the genetic screening of mutants is first to mutate seeds through chemical (EMS), physical (fast neutron) or T-DNA insertional (activation tagging) mutagenesis, and then identify the mutants with expected phenotypes in M1/T1 or M2/T2 generation [28]. Plants with dominant mutation display phenotypes in M1 generation, and recessive mutations in M2 generation. However, most of the mutations cause no evident phenotypic changes due to genetic redundancy or inappropriate screening conditions [9]. Here, we found three fertility restored lines in the M1 generation, named *satmf1*, *satmf2* and *satmf3* (Figure 2A and Supplementary Figure S3A), suggesting that these mutants are caused by dominant mutations. To rule out the possibility of seed contamination, we firstly performed the genotyping analysis by using *atm* specific primers (Supplementary Table S1), and found that *satmf1~3* are in *atm* mutant background (Supplementary Figure S3B). In order to further confirm their genetic backgrounds, the PCR products were mixed and used for sequencing, and we found, indeed, that *satmf1~3* are in an *atm* background (Supplementary Figure S3C,D). Therefore, the homozygous mutants of *satmf1~3* were used for the subsequent phenotypic analysis.

### 2.3. SATMF Suppresses the Early Senescence Phenotypes of atm Mutant and Improves Its Fertility

To evaluate the effects of SATMF on restoring *atm* fertility and leaf senescence, we analyzed silique length, number of seeds per silique, the size of seeds, and rosette leaves of *atm* and *satmf1~3*. Gain-of-function of SATMF suppressed the early flowering phenotype of *atm* mutant (Figure 2A), and *satmf1~3* plants had longer siliques (Col-0, 10.46 ± 1.33 mm; *atm*, 7.05 ± 1.09 mm; *satmf1*, 10.44 ± 0.93 mm; *satmf2*, 10.23 ± 1.68 mm; *satmf3*, 10.56 ± 1.62 mm) (Figure 2B,C), and more seeds per silique (Col-0, 42.68 ± 6.32; *atm*, 7.83 ± 4.8; *satmf1*, 41.46 ± 9.13; *satmf2*, 42.23 ± 5.58; *satmf3*, 42.54 ± 8.45) (Figure 2D), indicative of the restored fertility. As expected, the premature leaf senescence of the *atm* mutant was repressed in *satmf1~3* (Figure 2E) with higher levels of PSII Photochemical efficiency (Fv/Fm) and chlorophyll contents (Figure 2F,G). The above data suggest that the gain of function of SATMF effectively restores the abnormal traits of *atm* mutant.

Although *Arabidopsis* is traditionally regarded as a key model organism for plant biology, it is increasingly clear that *Arabidopsis* is an important tool for us to understand the molecular mechanisms underlying human diseases [25,29]. A comparison of the annotated *Arabidopsis* and human genome sequences shows that there is also a high proportion of genes related to human diseases in *Arabidopsis* [25], including ATM (Supplementary Table S2). Although *Arabidopsis* and humans diverged 1.6 billion years ago, relatively recent studies have shown that protein functions and cellular processes are unexpectedly conserved between these distant species [30]. The use of *Arabidopsis* has elucidated many discoveries directly related to human health and disease [29]. In particular, *Arabidopsis* has been used to dissect cellular processes related to neurodegenerative diseases such as Alzheimer’s disease, Parkinson’s disease, and Friedrich ataxia [29]. The molecular cloning and functional analysis of SATMF will help to understand the potential regulatory mechanism of ATM in plants, and may also provide a reference for developing new treatments for the disease Ataxia-telangiectasia (A-T).

### 2.4. Gain-of-Function of SATMF1~3 Restores the DSB Repair Efficiency in atm Background and Delays DSBs-Induced Leaf Senescence

**Figure 3:**
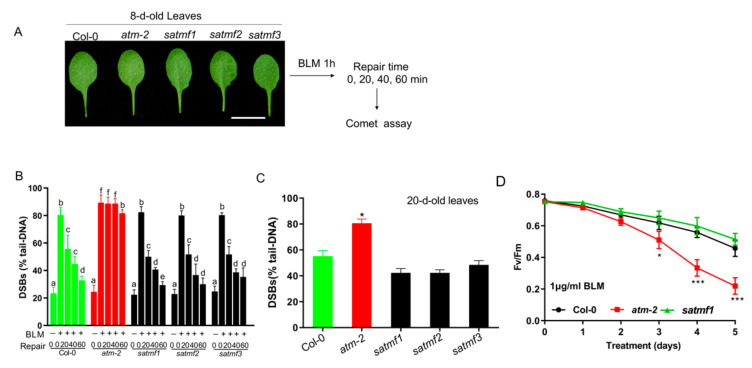
SATMF restores the DSB repair efficiency in *atm-2* background and delays DSBs-induced leaf senescence. (**A**) Experimental schematic of evaluation of DNA repair ability in *satmf1~3* by comet assay. The 8-d-old third and fourth leaves of Col-0, *atm-2* and *satmf1~3* upon treatment with 10 μg/mL BLM for 1 h were allowed to repair for 0, 20, 40, or 60 min, respectively, and used for comet assay. (**B**) Comet assay of the repair kinetics of the DSBs caused by BLM in the leaves in (**A**). Mean percentage of DNA in the tail at various recovery times after treatment was examined by comet assay. Three biological replicates were performed (mean ± S.D.). Different letters represent significant differences (two-way ANOVA and post-hoc Tukey test, *p* < 0.05). (**C**) Comet assay of DSBs levels in the third and fourth leaves of 20-d-old Col-0, *atm-2* and *satmf1~3* plants, evaluated as the mean percentage of DNA in the comet tail. Values shown represent the mean of three independent trials ± S.D. Student’s *t*-test, * *p* < 0.05 (**D**) Photochemical efficiency of PSII (Fv/Fm) in the leaves of Col-0, *atm-2*, *satmf1* plants upon treatment with 1 μg/mL BLM for up to 5 d. Values shown represent the mean of three independent trials ± S.D. Student’s *t*-test: * *p* < 0.05; *** *p* < 0.001.

The above-reported data demonstrate dominant mutations of SATMF1~3 significantly improve the fertility compared with *atm* mutant, which prompted us to examine whether SATMF1~3 restored the DNA repair capacity in *atm* background. To this end, a comet assay was performed in 8-d-old third and fourth leaves of Col-0, *atm* and *satmf1~3* plants before and after treatment with bleomycin (BLM) (Figure 3A), an inducer of DSBs [31]. A time-course analysis of DNA repair kinetics revealed that BLM treatment led to a sharp increase in DSBs that were efficiently repaired in 8-d-old leaves of Col-0 and *satmf1-3* but not in *atm* mutant (Figure 3B), suggesting that DNA repair capacity for DSBs was efficiently restored by the gain of SATMF1~3 function. To confirm this observation, we detected the DSBs levels in 20-d-old leaves of Col-0 and *satmf1~3* mutants. Accumulation of DSBs was evidently increased as leaves aged compared with 8-d-old leaves in Col-0 (*p* < 0.05). Higher levels of DSBs were found in 20-d-old leaves of *atm* in comparison with that in the leaves of Col-0 and *satmf1~3* (*p* < 0.05), while no significant difference was observed between Col-0 and *satmf1~3* mutant (Figure 3C), suggesting that SATMF1~3 efficiently suppress age-induced accumulation of damaged DNA. 

To investigate the roles of SATMF1~3 in DSB-induced leaf senescence, the detached leaves of Col-0, *atm* and *satmf1~3* were treated with 1 μg/mL BLM. The decline in the photochemical efficiency of PSII (Fv/Fm) upon treatment with BLM in *atm* leaves was more pronounced than that in Col-0 and *satmf1* leaves (Figure 3D). A slightly but non-significantly higher level of Fv/Fm was observed in *satmf1* compared to Col-0 at the same time points. These data suggest that SATMF1 plays an important role in suppressing DSB-induced leaf senescence. 

### 2.5. The ATM Loss-of-Function Mutant is Useful for Functional Genomics Research in Arabidopsis

**Figure 4:**
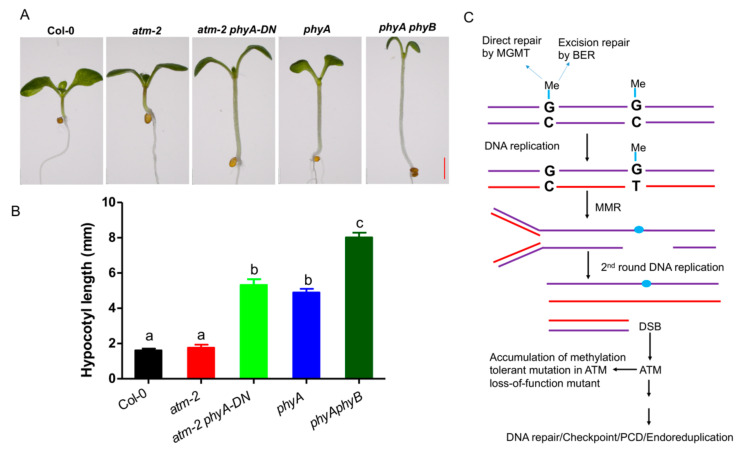
ATM loss-of-function mutants can be used to produce genetic materials in *Arabidopsis*. (**A**) Identification of a dominant negative (DN) mutant *phyA-DN* in EMS-treated *atm* mutant. After sowing, the petri dish with the seeds is placed at 4 °C for 3 days, and cultivated in a greenhouse at 22 °C for 5 days (18 L/6 D). (**B**) Measurement of hypocotyl length of the indicated seedlings. Different letters represent significant differences (two-way ANOVA and post-hoc Tukey test, *p* < 0.05). (**C**) Schematic representation of usage of *atm* for generating genetic materials.

During the process of screening suppressors, we not only obtained the fertility-restored suppressors of *atm* mutant, but also found that the M1 plants derived from mutagenized *atm* seeds displayed more abundant phenotypes than that of Col-0. One of the mutants displayed longer petiole and hypocotyl than *atm*, reminiscent of *Arabidopsis* phytochrome (*phy)*-related mutants (Figure 4A,B). Sequencing analysis of genotyping PCR product revealed that a point mutation occurred in *PHYA* gene (Supplementary Figure S4), leading to a dominant negative (DN) phenotype (Figure 4A,B). We used these seeds to screen for ethylene-insensitive mutants and showed that more mutants were obtained in *atm-2* background. 

Given that ATM is an important component in DNA repair, DNA damage caused by alkylating agents such as EMS will cause more mutation in ATM loss of function mutant [32]. Damaged DNA can be repaired through multiple pathways, including direct repair by methylguanine methyl transferase (MGMT) and BER, or MMR (Figure 4C). If unrepaired, the methylguanine (MeG) preferably mispairs with T during DNA replication leading to transitions from G:C to A:T (Figure 4C) [32]. Phosphorylation of ATM activates MeG-dependent DNA damage response. MeG lesions induce sister chromatid exchanges (SCE) and chromosomal aberrations via MMR-dependent pathway in the second cell cycle [32]. The gaps and nicks are generated during this phase and can form DSB that are handled by ATM-mediated homologous repair. While loss of ATM function, cells become “methylation-tolerant” accumulating mutations and escape cell death in the presence of unrepaired MeG, leading to generation of seeds with more mutated sites (Figure 4C). Collectively, our data suggest that *atm* mutant is a valuable tool for functional genomics research.

## 3. Materials and Methods

### 3.1. Plant Materials and Growth Conditions

All the *Arabidopsis* plant materials used in this study were in the Col-0 background. Plants were grown in an environmentally controlled growth room under long-day conditions (16 L/8 D) at 22 °C and 65% relative humidity.

### 3.2. Plasmid Construction and Generation of Transgenic Plants

To generate *35S:GFP-ATM*/Col-0, the full-length *ATM* CDS sequence was amplified and then inserted into pEGAD vector [33]. To construct *ProATM:GUS*/Col-0, a 3 kb genomic promoter sequence was amplified and inserted into pBI101 vector [34]. The amplified fragments were inserted into respective vectors by using the in-fusion enzyme (TAKARA, Shiga, Japan). All constructs were transformed into *Agrobacterium tumefaciens* cells (strain GV3101) which was used to transform Col-0 plants by the floral dip method [35]. All primer sequences used here are listed in Supplementary Table S1.

### 3.3. Assays of Age-Dependent and Bleomycin-Induced Leaf Senescence

To assess age-dependent leaf senescence, third and fourth rosette leaves were harvested from 40-d-old plants. For bleomycin (BLM)-induced leaf senescence, 8-d-old third and fourth rosette leaves were detached and floated on 3 mM MES buffer (pH 5.7) supplemented with or without 1 μg/mL BLM for up to 5 d under long-day conditions. Photochemical efficiency of photosystem II (Fv/Fm) was measured using a MultispeQ (East Lansing, MI, USA) instrument, and chlorophyll contents were measured using a chlorophyll meter SPAD502 Plus (Konica Minolta, Hino-shi, Tokyo, Japan).

### 3.4. EMS Mutagenesis of Arabidopsis Seeds

Ethyl methanesulfonate (EMS) mutagenesis of *atm* seeds was performed in the laboratory of Prof. Hong Gil Nam (DGIST, Korea) according to protocols as described previously [24]. Given that *atm* mutant is more sensitive to EMS than wild-type Col-0 (Supplementary Figure S2C), seeds of Col-0 and *atm* were treated with EMS with a final concentration of 0.2% (1.5 g, ~75,000 seeds), 0.3% (2 g, ~100,000 seeds) and 0.4% (1 g, ~50,000 seeds) for 15 h, 12 h and 10 h respectively. M1 seeds were sown in soil uniformly with the use of a squeeze bottle and used for direct screening for dominant mutants, and M2 seeds were subjected to screen for recessive mutants. 

### 3.5. Comet Assay of DSBs

DSB levels were determined using a comet assay with a neutral protocol, measuring the amount of DNA in the tail, as described previously [36]. Images of comets were captured at 20-fold magnification using a monochrome CCD camera. Images were analyzed with the Image J software plugin OpenComt (http://www.cometbio.org/) [37]. Each experimental point is represented by the mean value from three replicates of electrophoresis gels.

### 3.6. RNA Isolation and Real-Time PCR

Total RNA was isolated using plant RNA kits (ER301; TransGen Biotech, Beijing, China), and complementary DNA was produced with cDNA Synthesis kit (AT341; TransGen Biotech) according to the manufacturers’ protocols. Transcript levels were assessed by qPCR kit (AQ111; TransGen Biotech) using Applied Biosystems 7500 Real-Time PCR System (Thermo Scientific, Waltham, MA, USA). The primers used in this study are listed in Supplementary Table S1.

## 4. Conclusions

Previous results demonstrated that ATM functions as a negative regulator of plant aging through controlling age-induced DSBs accumulation, which has significant implications for the conservation of aging mechanisms in animals and plants [12]. We obtained three suppressors of *atm* mutant with delayed senescence and restored fertility. Functional analysis of SATMF will help to understand the underlying regulatory mechanism of ATM in plants, and may also provide a reference for developing new treatments for the disease Ataxia-telangiectasia (A-T). A-T is a neurodegenerative disease [38], and no curative medication is found to effectively treat this disease [39]. Although *Arabidopsis* is traditionally viewed as the key model organism for plant biology, it is becoming gradually clear that *Arabidopsis* represents an invaluable tool for understanding molecular mechanisms underlying human diseases [29]. Additionally, our data also show that *atm* mutant might be a valuable tool for functional genomics research in *Arabidopsis*.

## Supplementary Materials

Supplementary materials can be found at https://www.mdpi.com/1422-0067/21/21/8120/s1.

## Abbreviations

ATM	Ataxia Telangiectasia Mutated
ATR	ATM- and RAD3-related
DSB	Double-strand Break
SATMF	Suppressor of *atm* in Fertility
BER	Base Excision Repair
HR	Homologous Recombination
NHEJ	Non-Homologous End Joining
EMS	Ethyl MethaneSulfonate
