# Supplementary material for: SATMF Suppresses the Premature Senescence Phenotype of the ATM Loss-of-Function Mutant and Improves Its Fertility in Arabidopsis
Source: Int J Mol Sci. 2020 Oct 30;21(21):8120. doi: 10.3390/ijms21218120 (PMC7662627; doi:10.3390/ijms21218120)
Supplement: Supplementary file 1 [file ijms-21-08120-s001.pdf]

Supplementary Materials

SATMF Suppresses the Premature Senescence Phenotype of the ATM Loss-of-Function Mutant and Improves Its Fertility in *Arabidopsis*

Yi Zhang, Hou-Ling Wang, Yuhan Gao, Hongwei Guo and Zhonghai Li

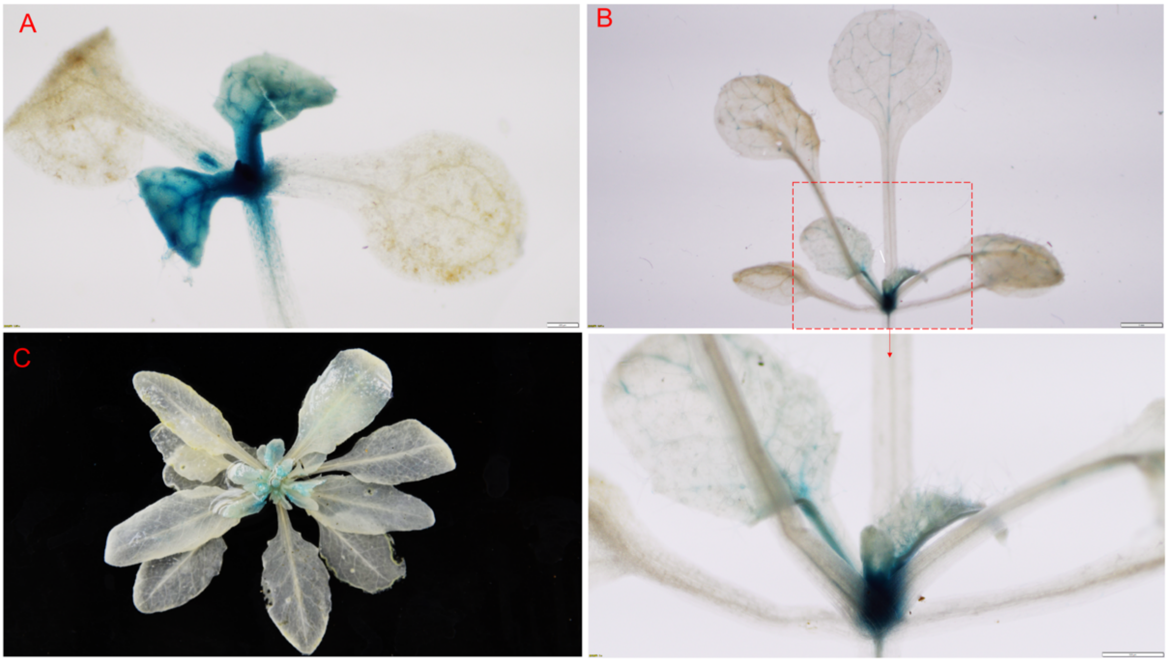

Figure S1. Histological GUS staining analysis of gene expression of ATM at different developmental stages. (**A**) Rosette leaves of 8-day-old seedling of *pATM-GUS*/Col-0. (**B**) Rosette leaves of 16-day-old plant of *pATM-GUS*/Col-0. (**C**) Rosette leaves of 48-day-old plant of *pATM-GUS*/Col-0.

A

Phenotypes of *atm-2* mutant
- ✓ Early flowering
- ✓ Early senescence
- ✓ Decreased fertility
- ✓ Increased plant height
- ✓ Hypersensitive to DNA damage agents

Phenotypes of *suppressors* of *atm-2* mutant
- ✓ normal flowering
- ✓ normal senescence
- ✓ recused fertility
- ✓ Normal plant height
- ✓ DNA damage agents

B

Seeds	EMS	Time	Phenotype
1.5g/~75,000	0.20%	15 h	Similar to the untreated *atm-2* plants
2g/~100,000	0.30%	12 h	Slightly delayed germination compared with 0.2%
1g/~50,000	0.40%	10 h	Strongly delayed germination compared with 0.2% and 0.3%

C

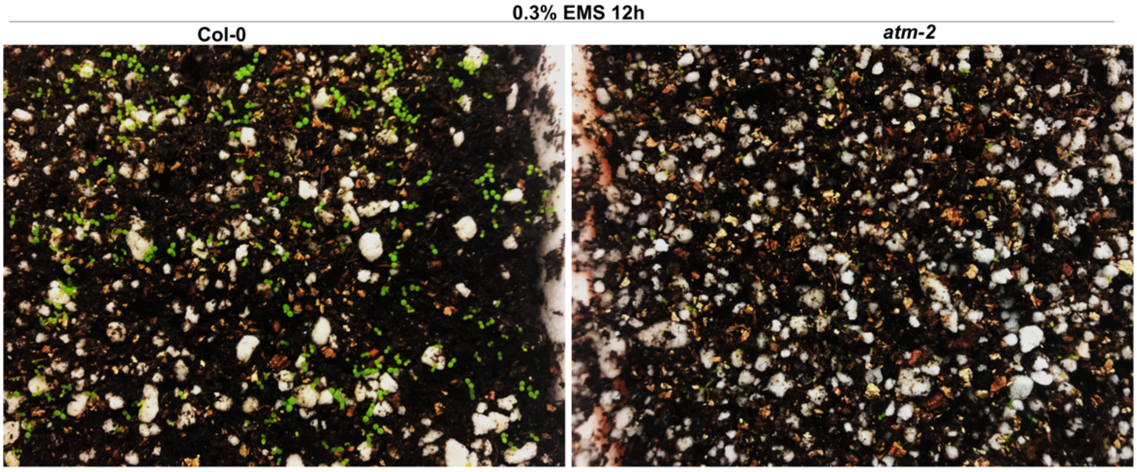

Figure S2. Screen for *suppressor of atm mutant in fertility* (*satmf*) by EMS. (**A**) Experimental design for screening the expected phenotypes of *stamf* mutants. (**B**) EMS mutagenesis of *atm-2* seeds by using different experimental conditions. (**C**) Seeds of *atm-2* mutant is hypersensitive to EMS in comparison to Col-0.

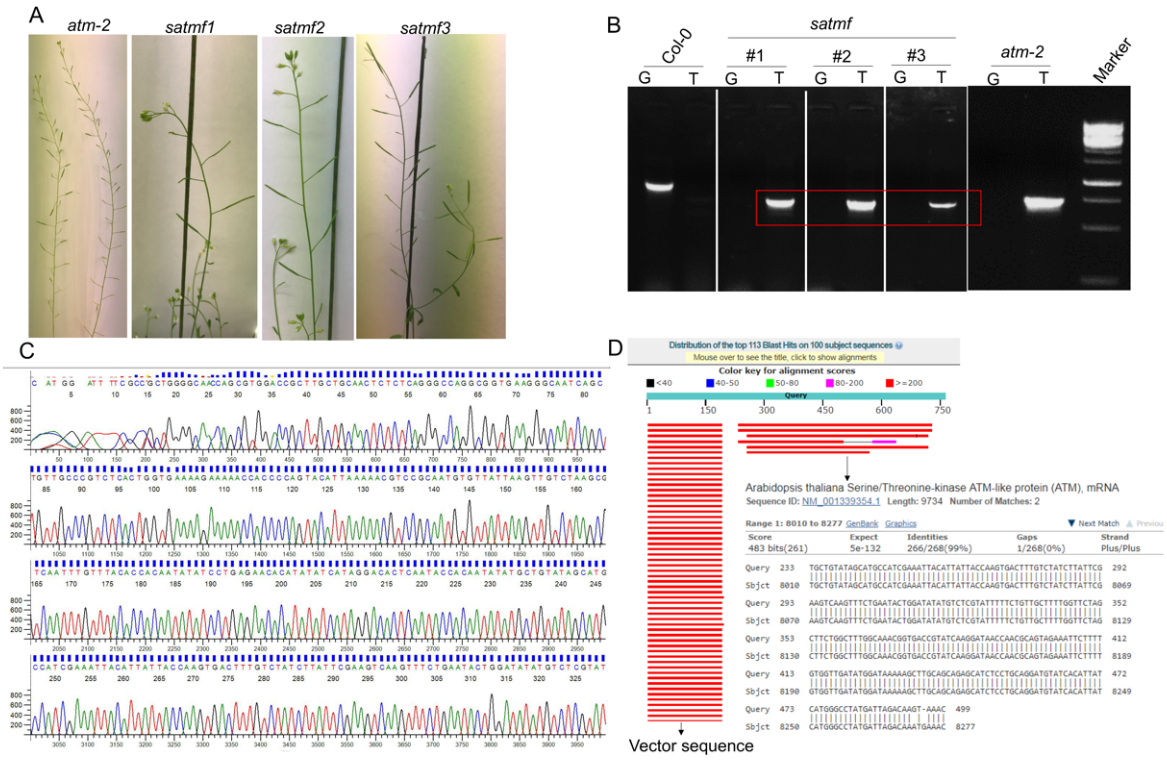

Figure S3. Molecular identification of *satmf* mutants. (**A**) Fertility of plants of Col-0, *atm-2*, *satmf1*, *satmf2* and *satmf3*. (**B**) Genotyping analysis of *atm-2*, *satmf1*, *satmf2* and *satmf3* plants. (**C**) Confirmation of the backgrounds of *satmf1*, *satmf2* and *satmf3* plants by sequencing analysis of PCR products in (**B**). The "G" PCR reaction tests for the ability to amplify a genome region that will be present in wild type and heterozygous lines, but will not amplify in homozygous lines. The "T-DNA" PCR reaction checks for the presence of a T-DNA insertion. (**D**) BLAST analysis of the sequencing data confirms the backgrounds of *satmf* mutants.

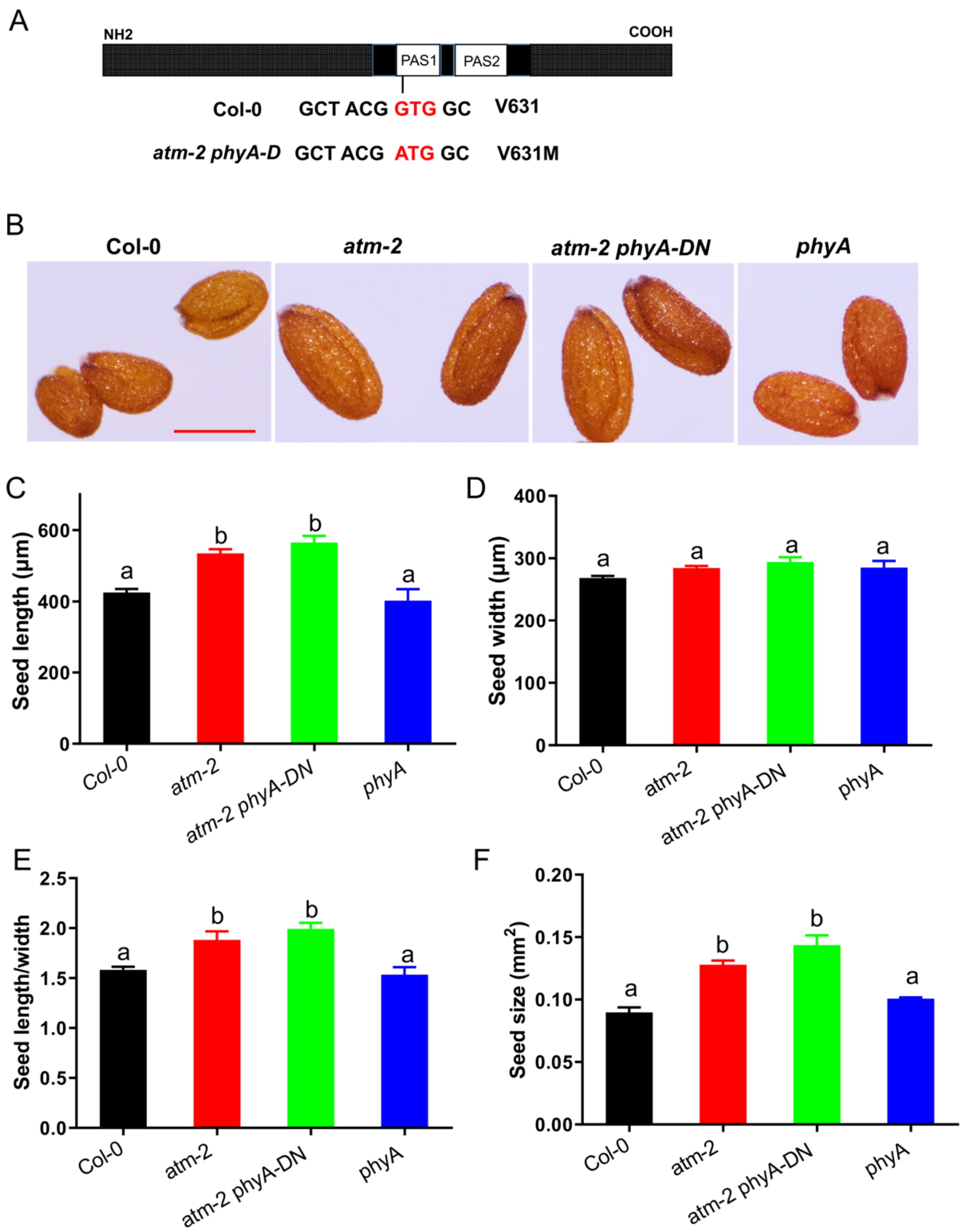

Figure S4. Identification of a dominant negative (*DN*) mutant of *phyA-DN*. (**A**) The structural features of *phyA* and the molecular lesion of the *phyA* mutation in *atm* background. There is a single amino acid residue mutation (V631M) in the translated proteins of *atm-2 phyA-DN* double mutant. (**B**) Mature seeds of Col-0, *atm-2*, *atm-2 phyA-DN* and *phyA* mutants. (**C**) Seed length, (**D**) seed width, (**E**) the ratio of length to width, and (**F**) seed area of mature dried seeds of Col-0, *atm-2*, *atm-2 phyA-DN* and *phyA* mutants. Values that differ at the 0.05 significance level are labeled with different letters.

Table S1. Primers used in this study.

Locus	Primer	Sequence (5′ to 3′)	Purpose
AT3G48190	ATM_F	ATGGTTGCTTCGAGGGATGTCC	Gene cloning
AT3G48190	ATM_R	CTAGCAAGTCCGATGCCAATTA	Gene cloning
AT3G48190	pATM_F	AGATCTTAGTCTAAAATCTATCC	Promoter cloning
AT3G48190	pATM_R	TGTGAGAGTGAGAGTAAGTGAG	Promoter cloning
AT3G48190	SALK_006953LP	ATCCATGTGGTTCAGTCTTGC	Genotyping
AT3G48190	SALK_006953RP	TTGGTATCCTGCAGAGGAAAG	Genotyping
	LB1.3	ATTTTGCCGATTTCGGAAC	Genotyping

Table S2. *Arabidopsis* genes with similarities to human disease genes (E value < 10^{-80}).

Human Disease Gene	E Value	Gene Code	Arabidopsis Hit
Darier–White, SERCA	5.9×10^{-272}	AT1G10130	ARABIDOPSIS THALIANA ER-TYPE CA2+-ATPASE 3 (AtECA3)
Xeroderma Pigmentosum, D-XPD	7.2×10^{-228}	AT1G03210	Putative DNA repair protein
Xeroderma pigment, B-ERCC3	9.6×10^{-214}	AT5G41360	DNA excision repair cross-complementing protein
Hyperinsulinism, ABCC8	7.1×10^{-188}	AT1G04120	Multidrug resistance protein
Renal tubul. acidosis, ATP6B1	1.0×10^{-182}	AT4G38510	Probable H+-transporting ATPase
HDL deficiency 1, ABCA1	2.4×10^{-181}	AT2G41700	Putative ABC transporter
Wilson, ATP7B	7.6×10^{-181}	AT5G44790	ATP-dependent copper transporter
Immunodeficiency, DNA Ligase 1	8.2×10^{-172}	AT1G08030	DNA ligase
Stargardt's, ABCA4	2.8×10^{-168}	AT2G41700	Putative ABC transporter
**Ataxia telangiectasia, ATM**	$\mathbf{3.1 \times 10^{-168}}$	**AT3G48190**	**Ataxia telangiectasia mutated protein AtATM**
Niemann–Pick, NPC1	1.2×10^{-166}	AT1G42470	Niemann–Pick C disease protein-like protein
Menkes, ATP7A	1.1×10^{-153}	AT1G63450	ATP-dependent copper transporter, putative
HNPCC, MLH1	1.5×10^{-150}	AT4G09140	MLH1 protein
Deafness, hereditary, MYO15	2.7×10^{-150}	AT2G31900	Putative unconventional myosin
Fam, cardiac myopathy, MYH7	6.5×10^{-147}	AT3G19960	Putative myosin heavy chain
Xeroderma Pigmentosum, F-XPF	1.4×10^{-146}	AT5G41150	AtRAD1 confers resistance to UV radiation. DNA repair
G6PD deficiency, G6PD	7.6×10^{-137}	AT5G40760	Glucose-6-phosphate dehydrogenase
Cystic fibrosis, ABCC7	2.3×10^{-135}	AT3G62700	MULTIDRUG RESISTANCE-ASSOCIATED PROTEIN 10
Glycerol kinase defic, GK	7.9×10^{-135}	AT1G80460	NONHOST RESISTANCE TO P. S. PHASEOLICOLA 1
HNPCC, MSH3	6.6×10^{-134}	AT4G25540	Putative DNA mismatch repair protein
HNPCC, PMS2	5.1×10^{-128}	AT4G02460	DNA mismatch repair,POSTMEIOTIC SEGREGATION 1
Zellweger, PEX1	4.1×10^{-125}	AT5G08470	Putative AAA-ATPases involved in peroxisome biogenesis
HNPCC, MSH6	9.6×10^{-122}	AT4G02070	G/T DNA mismatch repair enzyme
Bloom, BLM	4.4×10^{-109}	AT1G10930	DNA helicase ATRECQ4A involved in the maintenance of genome stability
Finnish amyloidosis, GSN	2.2×10^{-107}	AT5G57320	Villin 5, actin filament bundling protein
Chediak–Higashi, CHS1	5.8×10^{-99}	AT1G03070	Putative transport protein,Bax inhibitor-1 family protein
Xeroderma Pigmentosum, G-XPG	7.1×10^{-89}	AT3G28030	Non-photoreactive DNA repair, nucleotide-excision repair, response to UV-B
Bare lymphocyte, ABCB3	1.3×10^{-84}	AT5G39040	ABC transporter-like protein ABCB27, ALUMINUM SENSITIVE 1
Citrullinemia, type I, ASS	3.2×10^{-83}	AT4G24830	Argininosuccinate synthase-like protein invovled in urea cycle
Coffin–Lowry, RPS6KA3	5.2×10^{-81}	AT3G08720	Ribosomal-protein S6 kinase (ATPK19), positive regulation of translation
